# Metabolic Signatures of Cultured Human Adipocytes from Metabolically Healthy versus Unhealthy Obese Individuals

**DOI:** 10.1371/journal.pone.0093148

**Published:** 2014-04-02

**Authors:** Anja Böhm, Anna Halama, Tobias Meile, Marty Zdichavsky, Rainer Lehmann, Cora Weigert, Andreas Fritsche, Norbert Stefan, Alfred Königsrainer, Hans-Ulrich Häring, Martin Hrabě de Angelis, Jerzy Adamski, Harald Staiger

**Affiliations:** 1 Helmholtz Zentrum München, German Research Center for Environmental Health, Institute of Experimental Genetics, Genome Analysis Center, Neuherberg, Germany; 2 Department of Internal Medicine IV, Division of Endocrinology, Diabetology, Angiology, Nephrology, and Clinical Chemistry, University Hospital, Eberhard Karls University, Tübingen, Germany; 3 German Center for Diabetes Research, Neuherberg, Germany; 4 Institute for Diabetes Research and Metabolic Diseases of the Helmholtz Center München at the Eberhard Karls University of Tübingen, Tübingen, Germany; 5 Department of General, Visceral and Transplant Surgery, University Hospital, Eberhard Karls University, Tübingen, Germany; 6 Chair of Experimental Genetics, Technical University München, Freising-Weihenstephan, Germany; University of Catanzaro Magna Graecia, Italy

## Abstract

**Background and Aims:**

Among obese subjects, metabolically healthy and unhealthy obesity (MHO/MUHO) can be differentiated: the latter is characterized by whole-body insulin resistance, hepatic steatosis, and subclinical inflammation. Aim of this study was, to identify adipocyte-specific metabolic signatures and functional biomarkers for MHO versus MUHO.

**Methods:**

10 insulin-resistant (IR) vs. 10 insulin-sensitive (IS) non-diabetic morbidly obese (BMI >40 kg/m^2^) Caucasians were matched for gender, age, BMI, and percentage of body fat. From subcutaneous fat biopsies, primary preadipocytes were isolated and differentiated to adipocytes in vitro. About 280 metabolites were investigated by a targeted metabolomic approach intracellularly, extracellularly, and in plasma.

**Results/Interpretation:**

Among others, aspartate was reduced intracellularly to one third (p = 0.0039) in IR adipocytes, pointing to a relative depletion of citric acid cycle metabolites or reduced aspartate uptake in MUHO. Other amino acids, already known to correlate with diabetes and/or obesity, were identified to differ between MUHO's and MHO's adipocytes, namely glutamine, histidine, and spermidine. Most species of phosphatidylcholines (PCs) were lower in MUHO's extracellular milieu, though simultaneously elevated intracellularly, e.g., PC aa C32∶3, pointing to increased PC synthesis and/or reduced PC release. Furthermore, altered arachidonic acid (AA) metabolism was found: 15(S)-HETE (15-hydroxy-eicosatetraenoic acid; 0 vs. 120pM; p = 0.0014), AA (1.5-fold; p = 0.0055) and docosahexaenoic acid (DHA, C22∶6; 2-fold; p = 0.0033) were higher in MUHO. This emphasizes a direct contribution of adipocytes to local adipose tissue inflammation. Elevated DHA, as an inhibitor of prostaglandin synthesis, might be a hint for counter-regulatory mechanisms in MUHO.

**Conclusion/Interpretation:**

We identified adipocyte-inherent metabolic alterations discriminating between MHO and MUHO.

## Introduction

Diabetes and obesity have developed to a worldwide problem of mankind, with immense financial and personal burden. The underlying pathomechanisms are not well understood, the prevention strategies are insufficient. Development of the metabolic syndrome demands the interplay of multiple organs, e.g., fat, liver, muscle, gut, and brain. Among these, the liver and in particular adipose tissue play a meaningful impact [1], [2]. Continuously, more and more adipokines are found to play a role in atherosclerosis, endothelial dysfunction, metabolic syndrome and diabetes.

In the obese state, several subphenotypes exist, e.g., metabolically healthy and unhealthy obesity (MHO/MUHO) [3], [4]; the latter includes whole-body insulin resistance, hepatic steatosis, and subclinical inflammation. Insulin resistance precedes type 2 diabetes for years, but the sequelae exert adverse effects from the beginning [5], [6]. There are several metabolomic studies searching for biomarkers of insulin resistance, obesity and glucose intolerance [7]–[16], but none of them are dealing with adipocyte-specific aspects. The aim of this study was, to carve out possible adipocyte-specific metabolic differences between MHO and MUHO, and to figure out novel adipocyte-related functional biomarkers leading to pathways discriminating MHO and MUHO. To accomplish this study, we applied targeted metabolomics.

## Methods

### Population

20 morbidly obese (BMI >40 kg/m^2^) subjects of Western European Descendent undergoing bariatric (gastric sleeve) surgery were selected. Based on insulin sensitivity index, subjects were divided into an IR and an IS group. Participants were matched for gender, age, BMI and percentage of body fat (see also table 1). Overt diabetes as well as other severe diseases (besides morbid obesity) and/or medication affecting glucose tolerance were exclusion criteria. All included participants underwent physical examination. Informed written consent was given by all individuals; the study protocol has been approved by the ethics committee of the university Tübingen and was in accordance with the declaration of Helsinki.

**Table 1:** Participants' clinical characteristics.

	IS	IR	p	p1	p2
N (women/men)	6/4	6/4	-	-	-
Age (y)	45±9	38±13	0.1	-	-
BMI (kg/m^2^)	52.5±8.9	51.1±6.9	0.7	1.0	-
Body fat (%)	48.0±12.1^a^	50.2±11.6	0.7	0.5	-
Lean Body Mass (kg)	76±8	74±8	0.8	0.4	0.2
AUC_Glucose 0–120_ (mmol/L)	16.5±2.3	18.1±3.0	0.2	0.0268	0.0256
Glucose_0_ (mmol/L)	5.5±0.1	5.7±0.2	0.6	0.2	0.2
Glucose_120_ (mmol/L)	7.3±1.3	7.5±	0.9	0.3	0.2
Insulin_0_ (pmol/L)	111.5±30.1	265.2±12.6	<0.0001	0.0002	<0.0001
Insulin_120_ (pMol/L)	468.8±227	1519.5±689.4	0.0003	0.0003	0.0003
HbA1c (%; mmol/mol)	5.6±0.4; 38±4.4^a^	6.0±0.3; 42±3.3^a^	0.0194	0.0304	0.0244
ISI OGTT (•10^6^ Lkg^−1^min^−1^)	7.04±2.25	2.55±0.64	<0.0001	<0.0001	<0.0001
Metabolic Syndrome (%)#	60	78	0.4	0.2	0.2
Waist circumference (cm)	135±5	133±5	0.9	0.8	0.8
Adipo-IR index	80±27	172±104	0.0017	0.0049	0.0004
RR_sys_ (mmHg)	123±17	131±13	0.2	0.2	0.2
RR_dia_ (mmHg)	80±12	84±9	0.3	0.2	0.2
Free fatty acids (μmol/L)	723±182^a^	651±207	0.4	0.7	0.8
Triglycerides (mg/dL)	154±85	177±90^a^	0.6	0.5	0.6
Total cholesterol (mg/dL)	216±55	198±35	0.5	1.0	1.0
HDL cholesterol (mg/dL)	53±14^a^	41±5^a^	0.0312	0.1	0.2
LDL cholesterol (mg/dL)	131±36^a^	134±27^a^	0.7	0.2	0.3
Leukocytes (μL^−1^)	6,895±1,792	10,459±3,102	0.0024	0.0103	0.0130
CRP (mg/dL)	0.62±0.54	1.42±1.00	0.2	0.06	0.06
GOT (U/L)	22±5^b^	25±10^a^	0.6	0.7	0.7
GPT (U/L)	23±9^b^	37±21^a^	0.1	0.3	0.3
γ-GT (U/L)	25±13^b^	50±60^a^	0.2	0.06	0.07
Fetuin-A (μg/mL)	335±213^a^	372±165	0.6	0.6	0.6
SHBG (nmol/L)	54.0±76.5^a^	27.6±9.19	0.3	0.3	0.3
PAI-1 (ng/mL)	90.4±28.9^a^	134.7±33.1	0.007	0.0024	0.0034
Leptin (ng/mL)	71.3±32.2^a^	95.7±97.4	0.6	1.0	1.0
Adiponectin (μg/mL)	9.17±2.61^a^	6.26±2.45	0.0391	0.07	0.08

Data represent number (N) or means ±SD. Prior to statistical analysis, data were log*_e_*-transformed in order to approximate normal distribution and adjusted; p =  unadjusted p-value; p1 =  p-value after adjustment for gender and age; p2 =  p-value after adjustment for gender, age, and BMI. # defined by criteria from International Diabetes Federation 2005 and American Heart Association 2005; ^a^ Available from only 9 subjects; ^b^ available from 8 subjects.

### Phenotyping

All participants underwent a 2 h 75 g OGTT, insulin sensitivity was calculated using the method of Matsuda and DeFronzo (10,000/square root of [fasting glucose x fasting insulin x mean glucose x mean insulin]) [17]. Routine laboratory tests were performed, partly by ELISAs (see table 1). In-depth-description is given in File S1.

### Isolation and culture of human adipocytes

Human subcutaneous fat biopsies were obtained during gastric sleeve surgery after overnight fast prior to surgery. Human primary preadipocytes were isolated as previously described [18]. Then, preadipocytes were grown up and subsequently differentiated into mature adipocytes over a 20-day culture period. Exact concentration and medium contents are described in ESM. Adipocytes from all donors were used in second pass. Differentiation success was checked microscopically by NileRed/DAPI- and OilRedO-staining (see exemplarily Figure S1). Moreover, density of OilRed- staining per well was estimated using the publicly available JPEG-plugin colorcounter.

### Sample Preparation

At day 20 of differentiation, sample collection was performed. For extracellular milieu, cell culture medium was collected exactly 48 h after the last medium exchange (6 wells were pooled to account for inter-well differences) and sterile filtered (0.2 μm; Corning, Wiesbaden, Germany). Where necessary, 0.001% butylated hydroxytoluene was added to prevent auto-oxidation of prostanoids. Thereafter, the samples were aliquotted and stored in a −80°C freezer until measurement. For cell lysates, the layer was washed twice with pre-warmed PBS; quenching and extraction was performed by cell scrapping in 80% methanol (pre-cooled to −20°C) and transferred to a tube, immediately cooled in liquid nitrogen. Prior to analysis, 3 wells were pooled and filled in a vial containing glass beads (VK-05; Peqlab, Erlangen, Germany). Homogenization took place in the Precellys24 (PeqLab) homogenizer at 4°C for three times over 30 seconds at 5500 rpm. After centrifugation, the supernatant was used for analysis.

### Metabolomic analysis

Adipocytes from the MHO and MUHO were characterized with targeted metabolomics. The p180 kit of Biocrates Life Sciences AG (Innsbruck, Austria) allows a simultaneous quantification of 186 metabolites, including free carnitine, 40 acylcarnitines (Cx:y), 21 amino acids, 19 biogenic amines, hexoses, 90 glycerophospholipids (14 lysophosphatidylcholines (lysoPC) and 76 phosphatidylcholines (PC)), and 15 sphingolipids (SMx:y). The abbreviations Cx:y are used to describe the total number of carbons and double bonds of all chains, respectively. The assay procedures of the Absolute*IDQ* ^™^ p180 kit as well as the metabolite nomenclature have been described in detail previously [19], [20]. Sample handling was performed by a Hamilton Microlab STAR^™^ robot (Hamilton Bonaduz AG, Bonaduz, Switzerland) and an Ultravap nitrogen evaporator (Porvair Sciences, Leatherhead, U.K.). Mass spectrometric (MS) analyses were done on a API 4000 LC/MS/MS System (AB Sciex Deutschland GmbH, Darmstadt, Germany) equipped with a 1200 Series HPLC (Agilent Technologies Deutschland GmbH, Böblingen, Germany) and a HTC PAL auto sampler (CTC Analytics, Zwingen, Switzerland) controlled by the software Analyst 1.5.1. Data evaluation for quantification of metabolite concentrations and quality assessment is performed with the MetIDQ software package, which is an integral part of the AbsoluteIDQ kit. Internal standards served as reference for the calculation of metabolite concentrations.

Further assays for free fatty acids, eicosanoids and oxidized fatty acids (prostaglandins), as well as energy metabolism intermediates were performed at Biocrates AG. Metabolites were measured by gas chromatography coupled with MS detection (for free fatty acids) or high performance liquid chromatography tandem MS with Multiple Reaction Monitoring (MRM) (for eicosanoids). For energy metabolites detection hydrophilic interaction liquid chromatography electrospray tandem MS method in highly selective negative MRM detection mode was used. Further details are given in File S1.

All values below detection limit (LOD) were set to zero, and metabolites with ‘zero’ or ‘not applicable’ in all individuals and/or with values ‘not applicable’ in more than 4 individuals of one group have been excluded. Regarding lysates, 129 metabolites remained. The multivariate analyses require complete datasets as well as same number of individuals for every single metabolite. Thus, the multivariate analyses of lysates only were performed with 82 metabolites, measured with p180 kit (table S1 in File S1). Concerning conditioned medium, 274 metabolites remained, and for multivariate analyses 121 metabolites (table S2 in File S1) were used.

For analysis in blood, 500 μl of EDTA (ethylenediaminetetraacetic acid) plasma were withdrawn from each participant after overnight-fasting and immediately frozen until measurement. The metabolomic analysis took place at Biocrates, Austria. Same analyses were performed as for the adipocytes.

Multiplex Assay (BioPlex Pro Human Cytokine 3-Plex-Assay, BioRad, Munich, Germany) was performed according to manufacturer's instructions to measure MCP-1, TNFα and IL-6. All metabolites and cytokines were normalized for the differentiation level of adipocytes, measured by percentage of OilRed staining per well (expressed as arbitrary units [AU]).

### Statistics

For all statistical analyses JMP 10.0 (SAS Institute Inc., USA) was used. Regarding multivariate analysis, Principal Component Analysis (PCA) was performed to reduce the magnitude of data and to identify groups [21]. To verify the discriminative potential of the metabolite subsets, a linear discriminant analysis (IR vs. IS) was used (Figures S2 and S3).

For further statistical analysis, an unpaired, two-sided t-test was performed, if normal distribution was given; either a hetero- or homoscedastic test was applied, depending on the equality of variances. The equality of variances was determined with 5 different tests (O'Brien, Brown-Forsythe, Levene, Bartlett, and two-sided F-Test) and assumed, if all tests didn't reject the null hypothesis. Non-parametric tests were performed as indicated. Correction for multiple testing was done with the Bonferroni procedure (p_Bonferroni_ = 1–0.95^1/n^). A p-value≤p_Bonferroni_ was considered to be statistically significant; intracellularly: p≤0.0004, extracellularly: p≤0.0002, ratios: p≤0.0029, plasma: 0.0002.

## Results

### Cell donors'clinical characteristics

Anthropometric and laboratory characteristics of the participants are summarized in table 1. 20 individuals from southwest Germany were matched for gender, age, BMI and percentage of body fat. Based on the selection process, there was a marked intergroup difference regarding the OGTT-derived whole-body insulin sensitivity. Although all participants were non-diabetic, a significant difference regarding HbA1c and AUC_Glucose_ could be detected. As expected, MUHO had higher inflammation markers (e.g., serum leukocytes), but there was no difference in liver parameters. Adiponectin levels were reduced in IR subjects, but did not reach significance. In contrast, a clear difference was seen in plasminogen activator inhibitor (PAI)-1, with higher concentrations (almost 1.5-fold; p = 0.0034) in the IR group.

There were no differences in degree of differentiation between IR and IS adipocyte cultures (p = 0.3).

### Intracellular differences between IR and IS in amino acids and PCs

To illustrate the plenty of data, a PCA was performed (Figure S4). Based on scree-plot (Figure S4A), the first three components were meaningful pertaining to PCA. As Eigen-values were decreasing rapidly, only the first two components with Eigen-values more than ten (Figure S4B) were taken into account for PCA (Figure S4C). Discriminant analysis showed good separation between the groups (Figure S2).

**Figure 1:**
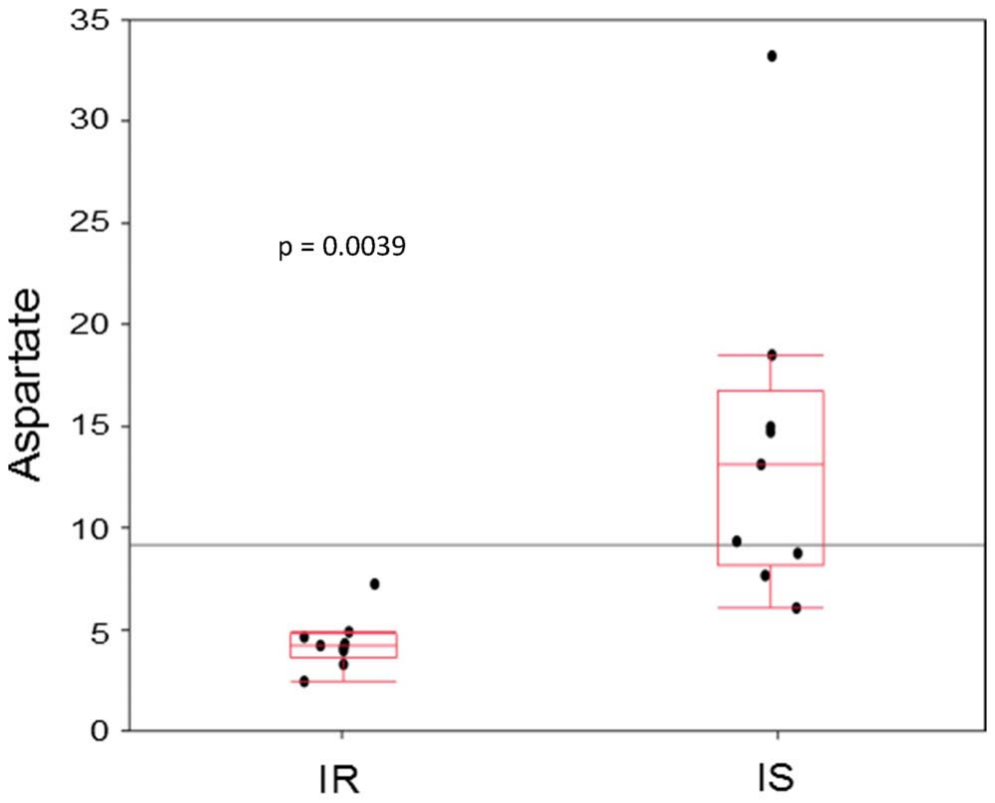
Aspartate. Intracellular levels in μM; IR vs. IS, n = 9; test: Wilcoxon. Horizontal line means grand mean.

Appropriate t-tests or non-parametric tests, respectively, revealed 12 metabolites with nominal differences (p<0.05) between groups (table 2): notably, aspartate was found to be decreased to one third in IR cells (Figure 1; p = 0.0039), which was replicated in another kit (p = 0.0097). These results partly resisted additional adjustment for age (p = 0.03 and 0.07). Other amino acids were elevated in IR (table 2), namely glutamine (p = 0.0070), histidine (p = 0.0226) and spermidine (p = 0.0161). Hexose (∼2.1-fold, p = 0.03) was higher in IR cells. Several PCs were affected, most species higher in IR adipocytes (table 2).

**Table 2:** Intracellular differences between IR and IS.

Assay	Metabolite	Test	N (IR vs IS)	p-value	IR
EMI	Aspartate	Wil	8 vs 10	0.0097	L
	Hexose	HE	9 vs 10	0.0383	H
p180	Aspartate	Wil	8 vs. 9	0.0039	L
	Glutamine	HO	8 vs. 9	0.007	H
	Histidine	HE	9 vs. 9	0.0226	H
	Spermidine	Wil	8 vs. 9	0.0161	H
	lysoPC C18∶0	HO	9 vs. 9	0.0321	H
	PC aa C32∶3	HE	9 vs. 9	0.0394	H *
	PC aa C34∶4	Wil	9 vs. 9	0.0218	H
	PC aa C36∶4	Wil	9 vs. 9	0.0325	H
	PC aa C36∶5	HO	9 vs. 9	0.0175	H
	SM C16∶1	HE	9 vs. 9	0.0499	H

Metabolites with nominal inter-group difference (p<0.05); EMI: Energy Metabolite Intermediates; HE =  heteroscedastic unpaired two-sided t-test, HO =  homoscedastic unpaired two-sided t-test, Wil =  Wilcoxon test, ME =  median test; p-value: p_Bonferroni_≤0.0004. H/L: metabolite-values in IR higher/lower vs. IS; PC: Phosphatidylcholine, aa/ae: acyl/ether side chain; SM: sphingolipids. * contrarily regulated in extracellular milieu.

### Extracellular differences between IR and IS in fatty acids, eicosanoids, and PCs

Figure S5 shows the PCA of extracellular metabolites. Scree-plot (Figure S5A) and Eigen-values (Figure S5B) revealed the first two components being important performing the PCA (Figure S5C). These two components explain more than 35% of the variance of all 121 successfully measured metabolites (p180 kit). Also extracellularly, a good inter-group separation was confirmed by discriminant analysis (Figure S3).

Analyzed with the appropriate tests, there were 13 metabolites with nominal differences (table 3); among others, the saturated fatty acid C18∶0 (stearic acid; 22-fold; p = 0.0427) was lower in extracellular milieu of MUHO.

**Table 3:** Differences between IR and IS in extracellular milieu.

Assay	Metabolite	Test	N (IR vs IS)	p-value	IR
FFA	C18∶0 (stearic acid)	ME	9 vs. 10	0.0427	L
	C18∶3 (linoleic acid)	HO	9 vs. 10	0.0488	H
	C20∶4 (arachidonic acid)	HO	9 vs.10	0.0100	H
Eicosanoids	15-S-HETE	Wil	9 vs. 10	0.0014	H
	AA	Wil	9 vs. 10	0.0055	H
	**DHA**	**Wil**	**9 vs. 10**	**0.0033**	**H**
p180	Serotonin	HO	8 vs. 10	0.0166	H
	PC aa C32∶0	HO	8 vs.10	0.0370	H
	PC aa C32∶3	HO	8 vs. 10	<0.0032	L *
	PC aa C36∶6	ME	8 vs. 10	0.0257	L
	PC ae C34∶3	HO	8 vs. 10	0.0494	L
	PC ae C42∶2	HO	8 vs. 10	0.0059	H
	SM C20∶2	ME	8 vs. 10	0.0245	L
	SM C22∶3	ME	8 vs. 10	0.0455	H

Metabolites with nominal inter-group difference (p<0.05); tests: HE =  heteroscedastic unpaired two-sided t-test, HO =  homoscedastic unpaired two-sided t-test, Wil =  Wilcoxon test, ME =  median test; corrected p-values: p_Bonferroni_<0.0002. Asterisk (*) marks statistical significance after Bonferroni correction. PC: Phosphatidylcholine, aa/ae: acyl/ether side chain; SM: sphingolipids. **Bold**: also affected in plasma of our probands. * contrarily regulated intracellularly. H/L: metabolite-values in IR higher/lower vs. IS.

**Figure 2:**
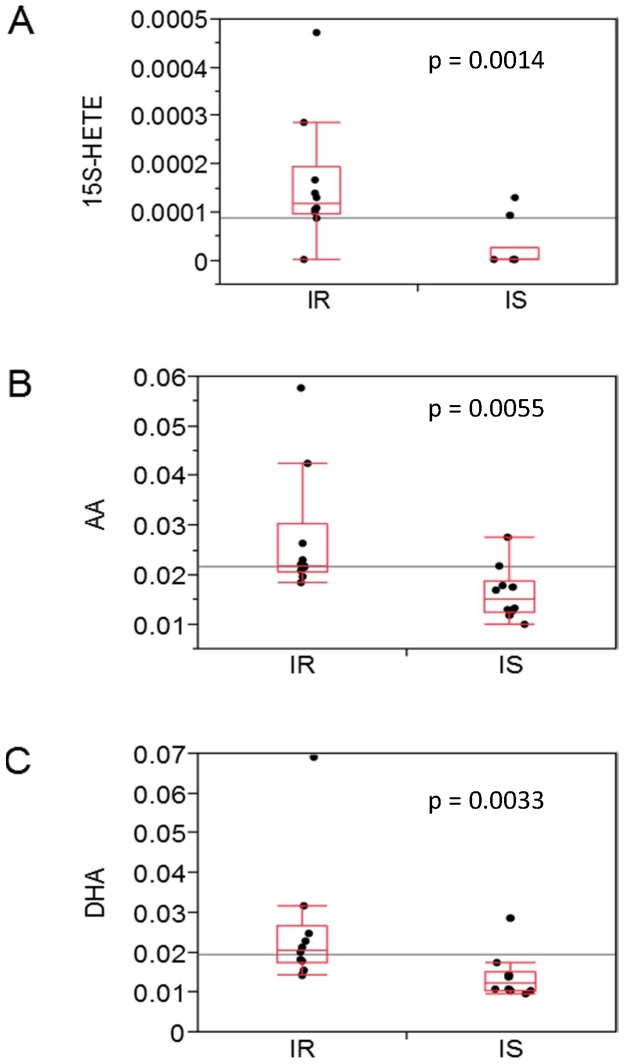
Arachidonic acid metabolism. Extracellular milieu; metabolite levels in μM; IR vs. IS; n = 10; (A) 15S-HETE: 15-hydroxy-eicosatetraenoic acid, median test; (B) AA: arachidonic acid, Wilcoxon; (C) DHA: docosahexaenoic acid (Wilcoxon). Horizontal line means grand mean.

**Figure 3:**
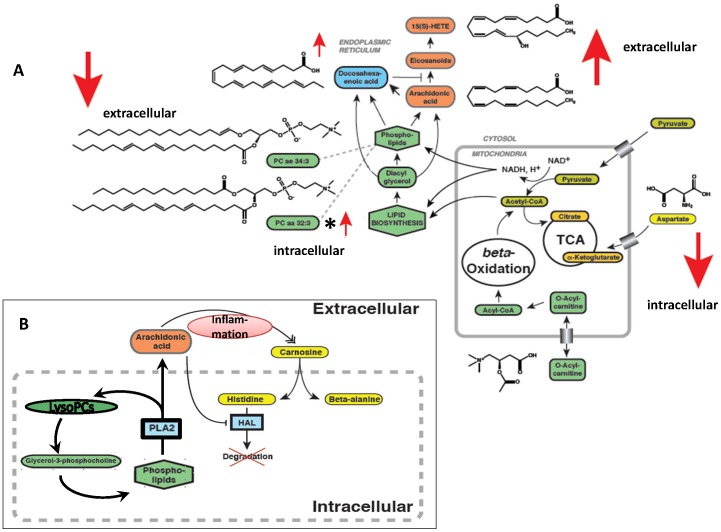
Overview on metabolite changes. Connecting processes like cofactors and intermediates are shown. Large arrows indicate change direction in the IR group. (A) Intracellular pathways; (B) Interactions between intracellular and extracellular metabolites; * Contrarily regulated intracellularly. HAL: histidine ammonia-lyase.

Furthermore, 15(S)-HETE (15-hydroxy-eicosatetraenoic acid; 0 vs. 120 nM; p = 0.0014), arachidonic acid (AA; 1.5-fold; p = 0.0055) and docosahexaenoic acid (DHA, C22∶6; 2-fold; p = 0.0033) emerged, all higher in IR (Figure 2), and all related to AA metabolism (Figure 3A, orange pathway). AA was confirmed also by the free fatty acid kit (p = 0.01). After additional adjustment for age, p-values decreased to: 15(S)-HETE (p = 0.08), AA (p = 0.12; second kit p = 0.02), and DHA (p = 0.02). PCaa32∶3 was reduced in IR (p = 0.0032), while elevated intracellularly (p = 0.0394).

Additionally, several ratios or sums of metabolites were investigated (see table S3 in File S1); thereof, two remained significant after correction for multiple testing (p_Bonferroni_≤0.0029): citrulline/ornithine-ratio, displaying the ornithine carbamoylphosphate transferase activity (mean IRvs.IS: 0.08vs.0.06; p = 0.0002); and ornithine/arginine-ratio (arginase activity), (mean IRvs.IS: 0.10vs.0.13; p = 0.00005). Measurements of cytokines showed elevated levels of IL-6 (p = 0.0275) in IS. MCP-1 (p = 0.2) revealed no difference between the groups. TNFα was below limit of detection. Though, there was no correlation of IL-6 with the OGTT-derived insulin sensitivity index or adipose insulin resistance index neither (r^2^ = 0.1; p = 0.2, 0.1, respectively).

### Correlation of metabolites with adipose insulin resistance index

To strengthen our results, correlation analyses were made for the revealed metabolites (see Figure S6). Intracellularly, we saw significant correlation with adipose insulin resistance index for, e.g., aspartate (negatively correlated; p = 0.0157, p = 0.0016 second kit), and, among others, PCaa32∶3 (positively correlated; p = 0.0354), that was found to be altered intra- as well as extracellularly.

### Plasma metabolite differences between IR and IS

Plasma samples of the study participants were analyzed. 16 nominal significant metabolites are listed in table 4. Some fatty acids, as well as lysophosphatidylcholines and phosphatidylcholines were different between the groups. Divergent from the extracellular milieu, DHA was reduced in plasma of IR, so it may not derive abundantly from adipocytes to play a systemic role.

**Table 4:** Differences between IR and IS in plasma.

Metabolite	p-value	IR
C16 (acylcarnitine)	0.0108	L
C18∶1 (acylcarnitine)	0.0479	L
C 22∶4 (adrenic acid)	0.0331	L
C 22∶6 (cervonic acid [ = DHA])	0.0449	L §
lysoPC C16∶0	0.0122	L ‡
lysoPC C16∶1	0.0150	L
lysoPC C18∶1	0.0095	L
lysoPC C20∶3	0.0281	L
lysoPC C20∶4	0.0372	L
PC ae 36∶4	0.0261	L
PC ae 36∶5	0.0295	L -
PC ae 38∶5	0.0194	L
PC ae 38∶6	0.0248	L -
SM C26∶0	0.0483	L
glutamic acid	0.0160	H
Hexosephosphate	0.0291	L

Metabolites with nominal difference (p<0.05) analyzed by t-tests. PC: Phosphatidylcholine, ae: acyl/ether side chain; SM: sphingolipids; n = 10 vs. 10; H/L: metabolite-values in IR higher/lower vs. IS; DHA =  docosahexaenoic acid; ‡ also found by [12], [13]; § contrarily found extracellularly.

## Discussion

Aim of this study was, to uncover new insights in adipocyte metabolism of MHO versus MUHO. For this purpose, and to find novel functional discriminating biomarkers in adipocytes, the metabolic differences between *in vitro* differentiated subcutaneous adipocytes of IR vs. IS morbidly obese individuals were analysed using a targeted metabolomics approach. With respect to the participants' serum parameters (table 1), some of the results were expected, i.e., the observed increase in levels of inflammation markers in IR subjects and a slightly elevated gamma-glutamyl-transferase level. Notably, there was no significant difference in fatty acids between the groups. The higher levels of PAI-I in the IR group was somewhat surprising, as plasma levels of this adipokine correlate very well with BMI [22], [23]. However, elevation of PAI-1 per se is in line with several studies that linked PAI-1 not only to hypofibrinolysis, but also to inflammation and increased cardiovascular risk [24], [25]. Our results indicate that it is independent of adipose tissue mass, as the groups were well matched for body adiposity. Certainly, this finding will need further replication in larger cohorts.

As expected, adiponectin levels were reduced in the IR subjects, but this difference did not reach statistical significance. This could be due to the strong association of adiponectin levels with adipocyte mass, which was equal in both groups. In addition, we did not find significant differences between the groups regarding liver markers (e.g. transaminases, fetuin-A, SHBG). This is in contrast to [16] and may be due to the limited sample size. To study the adipocyte-specific impact on MUHO, we used the cell culture system. This minimized the influence of other cell types, i.e., macrophages and other immune cells escorting adipose tissue.

Even though being an *in vitro* project with culture time of several weeks, *in vivo* phenotypes seem to be conserved, pointing to genetic/epigenetic termination. The analysis of fat cell metabolism of IR vs. IS subjects revealed prominent differences in glycerophospholipids and sphingomyelins (see Figure 3 for an overview on contributing metabolites). Extracellularly as well as in plasma, almost all PCs were reduced in MUHO, interestingly, in contrast to higher PCs (same species) in MUHO's intracellular milieu; this is possibly due to an increased endogenous PC synthesis and/or reduced PC release in IR adipocytes (see Figure 3A).

Furthermore, PCs, same like sphingomyelins, are components of cell membranes; a contribution of membrane fluidity and flexibility to metabolic diseases is known [26], i.e., accumulation of those with decreased saturation level resulted in improved cell membrane fluidity.

Extracellular reduction of an even-numbered saturated fatty acid in MUHO (stearic acid) might reflect the insulin resistance-induced decrease in lipogenesis. However, it may also be linked to an increased level of glycerophospholipids intracellularly, including lysoPC C18∶0 and PCaaC36, containing chains with different saturation level. The saturation variants in PCaaC36 suggest desaturation of stearic acid inside the cells and its incorporation into PCs. Desaturation can be catalyzed by SCD1 or FADS2 molecules, known to play a role in obesity [27] and inflammatory diseases [28].

As adipose tissue inflammation plays a major role in obesity-induced insulin resistance, the AA pathway, we found elevated extracellularly in IR, is very interesting. Albeit only nominally significant (Figure 2) for the single pathway member, multiple intermediates of the AA metabolism were elevated (Figure 3A, orange pathway): first, DHA, an ω-3 fatty acid, also called cervonic acid; as DHA is an inhibitor of prostaglandin synthase [29], this probably displays a counter-regulatory - albeit insufficient - mechanism of MUHO's adipocytes. Second, another altered PUFA was arachidonic acid (AA), a very important local inflammatory metabolite. The AA pathway is linked to several complications of the metabolic syndrome/diabetes, e.g. vascular disease, diabetic retinopathy and nephropathy [30], at least in cell culture systems or mammals. Of note, it's difficult to translate results from one species to another, as great species-specific differences in AA metabolism are known [30]. AA itself stimulates glucose uptake in 3T3-L1 adipocytes [31]. Also an adipogenic effect of AA is discussed [32]. On the other hand, AA reduces basal glucose uptake in adipocytes and, furthermore, insulin-dependent AA-uptake is reduced in adipocytes of obese subjects [33]. This could be another explanation for elevated AA levels in IR and needs further clarification. Release of arachidonic acid (AA) can be catalyzed by phospholipase A2 (PLA2) or phospholipase C. Higher activities of some types of PLA2 are positively associated to diabetes [34]. Another intermediate of the arachidonate metabolism was concordantly affected (please see Figure 3A, orange pathway): 15S-HETE (15S-hydroxyeicosatetraenoic acid), one of the fatty acids derived from AA oxidation. The first step towards leukotriene synthesis is catalyzed by lipoxygenases (LOXs) producing HPETEs (hydroxyperoxyeicosatetraenoic acids). The intermediates are biologically active per se and/or can serve as precursors for lipid mediators [30]. LOXs are expressed in adipose tissue and promote adipogenesis, at least in mice and cell lines (3T3-L1) [35]. In humans, little is known about the 12-/15-LOXs. Interestingly, 15S-HETE is known to act as an agonist of PPAR (peroxisome proliferator-activated receptor) δ and PPARγ [36], [37], two important regulators of lipid metabolism. 12-HETE, which was also higher in the IR group but did not reach nominal significance, was already shown to directly promote insulin resistance and inflammation in adipocytes [38]. The extracellularly elevated levels of pro-inflammatory molecules may stimulate intracellular accumulation of histidine (an anti-inflammatory molecule and an essential amino acid). This finding can be supported by [39], where anti-oxidative and anti-inflammatory protection of histidine and carnosine against diabetic deterioration were reported.

One biogenic amine found to differ between the groups was serotonin, elevated extracellularly in IR. Serotonin is a hormone and neurotransmitter with versatile effects: e.g., on brain, endothelial cells, smooth muscles, heart, gut and coagulation, etc., usually depending on the particular receptor. Until now, it was not discovered to differ systemically in lean/obese or healthy/diabetic individuals; our finding could imply altered autocrine/paracrine effects in adipocytes of IR subjects. Very recently emerged that, at least in rats, there is a functional system of serotonin synthesis, reuptake and receptor activation in visceral adipose tissue [40].

The intracellularly reduced aspartate levels in IR individuals (Figure 1), could, via aspartate aminotransferase, reflect an altered citric acid cycle with relative depletion in IR subjects, what was already speculated at least in muscle cells [41], [42]. Furthermore, also a reduced aspartate uptake from medium (concentration: 50 μM) by IR adipocytes has to be discussed. Ornithine carbamoylphosphate transferase (calculated by the ratio of citrulline to ornithine) was significantly elevated in IR subjects in our study, perhaps reflecting an increased urea cycle flux in IR adipocytes, supported by reduced intracellular aspartate, which also supplies the urea cycle. Besides, arginine metabolism is known to play a role in insulin-stimulated glycogen-synthesis [43].

To close the circle, our *in vitro* results were checked for systemic relevance *in vivo*. Plasma samples of corresponding probands revealed no congruency of several extracellular metabolites (tables 1, 2, and 3), indicating a local role of the described metabolites.

A comparison of our plasma metabolite results with a very recently published study by Floegel et al [44] looking for serum metabolites associating with incidence of type 2 diabetes, did not show analogies, perhaps indicating different pathomechanisms for MUHO as for diabetes per se. With reduced lysoPC C16∶0 in IR a bridge is built to metabolically malign fatty liver [12], confirming the complex interaction between multiple organs regarding the pathogenesis of metabolic syndrome. Furthermore, lower lysoPC C20∶4 levels in IR are in good agreement with recent studies [8], [13], where serum metabolites in diabetic vs. non-diabetic participants were measured, and lysoPC C20∶4 was negatively associated with diabetes.

The systemically reduced DHA in IR reflects the decrease of beneficial ω3-FAs in IR and enforces our hypothesis of local (adipocyte-specific) counterregulatory mechanisms.

Of course, there are several limitations of this study: due to study design, we are not able to differentiate between cause and consequence of IR and/or elevated blood glucose, but the goal of this study was to find hints for novel adipocyte-related pathways and functional biomarkers. These biomarkers for MUHO/MHO of course have to be validated in larger but appropriate cohorts. Furthermore, our groups are not matched for daily eating habits; FA composition of tissues is at least partially influenced by diet, but we believe that this effect is negligible in morbidly obese subjects, who usually consume high-caloric diet. There is upcoming evidence that there are important sex differences regarding metabolism, but our study unfortunately does not have the power to discriminate between genders. Insulin sensitivity of our probands was estimated by an OGTT-derived index, not measured by clamps. Lastly, the findings result from an in vitro situation. But the differences sustained despite a several weeks lasting culture period. Thus, our findings reflecting insulin sensitivity are probably due to epigenetic long-term alterations in the adipocytes.

To conclude, this study reveals novel insights into the adipocyte-specific impact on IR. Several differences in cell membrane components, amino acids and fatty acids emerged. Alteration of the arachidonic acid metabolism points to a – albeit insufficient – counterregulation with negative feedback mechanism of MUHO's adipocytes.

## Supporting Information

Figure S1 **In vitro differentiated adipocytes.**
(TIFF)

Figure S2 **Discriminant Analysis of intracellular metabolites.**
(TIFF)

Figure S3 **Discriminant Analysis of extracellular milieu.**
(TIFF)

Figure S4 **Graphical inter-group description intracellularly.**
(TIFF)

Figure S5 **Graphical inter-group description extracellularly.**
(TIFF)

Figure S6 **Correlation of metabolites with AdipoIR index.**
(TIFF)

File S1 **File includes Supplementary Research Design and Methods, Supplemental References, legends for Figures S1-S6, and Tables S1–S3.**
(DOCX)
