# Supplementary figures and images for: Metabolic Signatures of Cultured Human Adipocytes from Metabolically Healthy versus Unhealthy Obese Individuals
Source: PLoS One. 2014 Apr 2;9(4):e93148. doi: 10.1371/journal.pone.0093148 (PMC3973696; doi:10.1371/journal.pone.0093148)

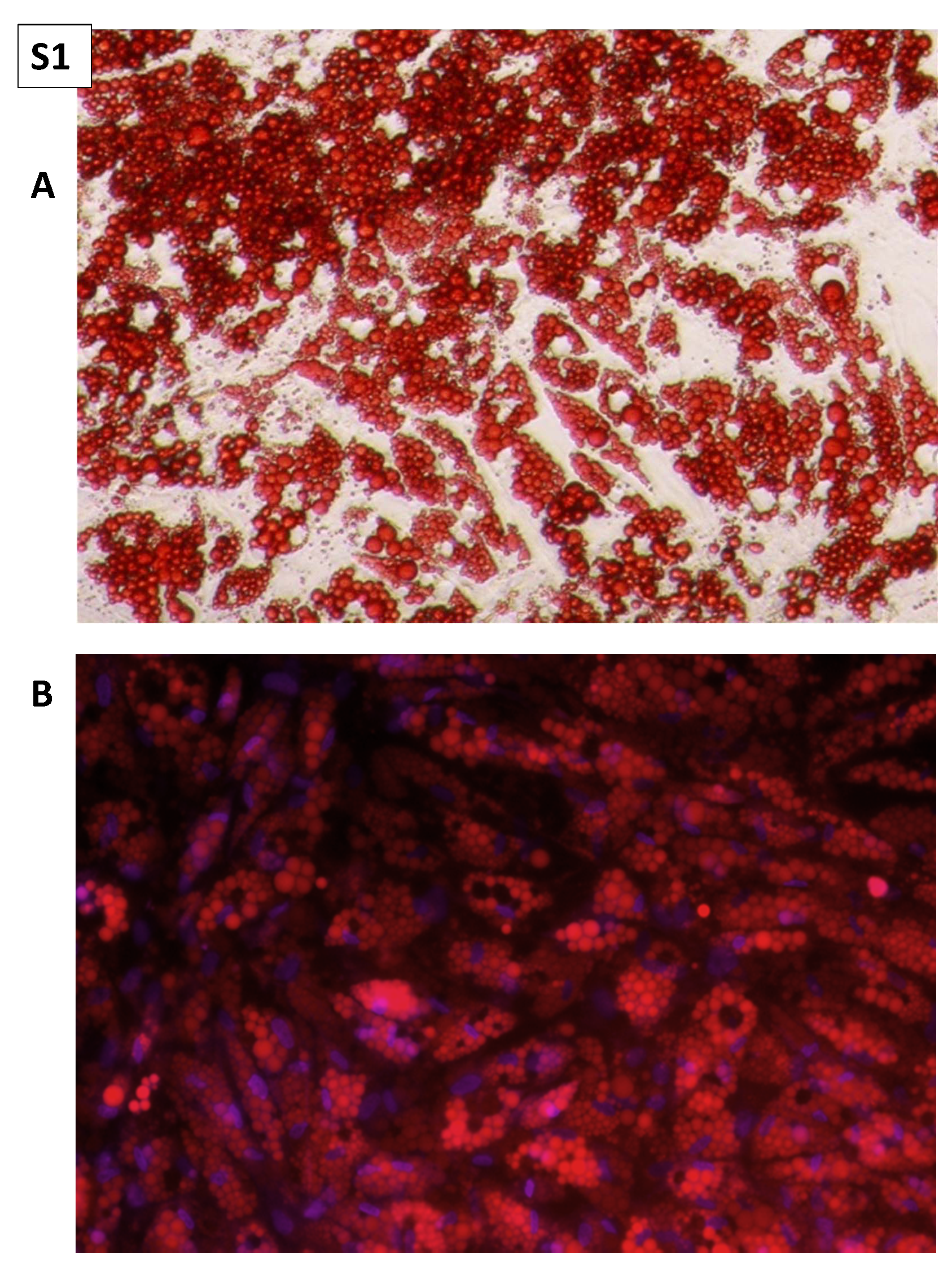

Supplement: Figure S1 — In vitro differentiated adipocytes. (TIFF) [file pone.0093148.s001.tiff]

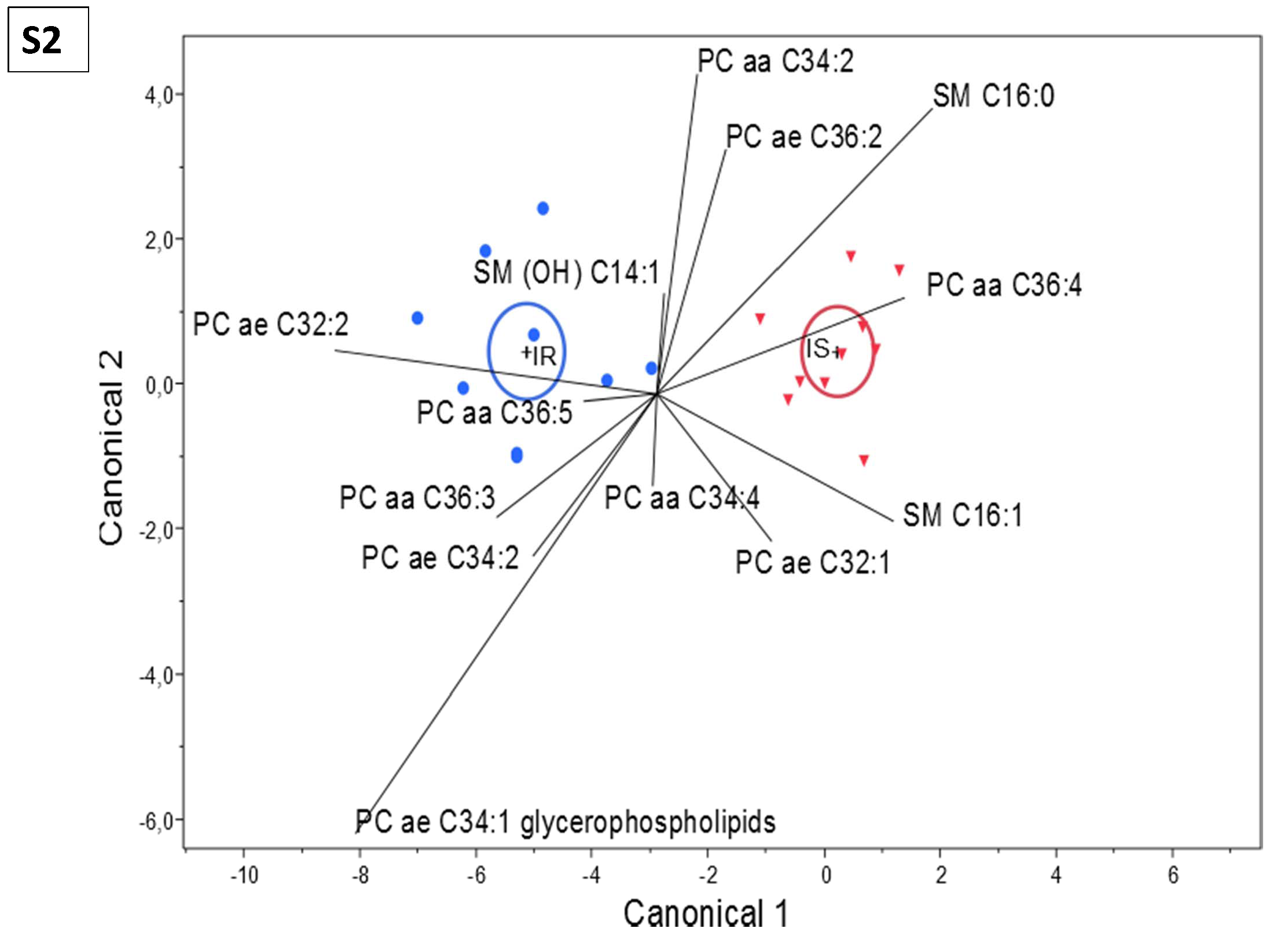

Supplement: Figure S2 — Discriminant Analysis of intracellular metabolites. (TIFF) [file pone.0093148.s002.tiff]

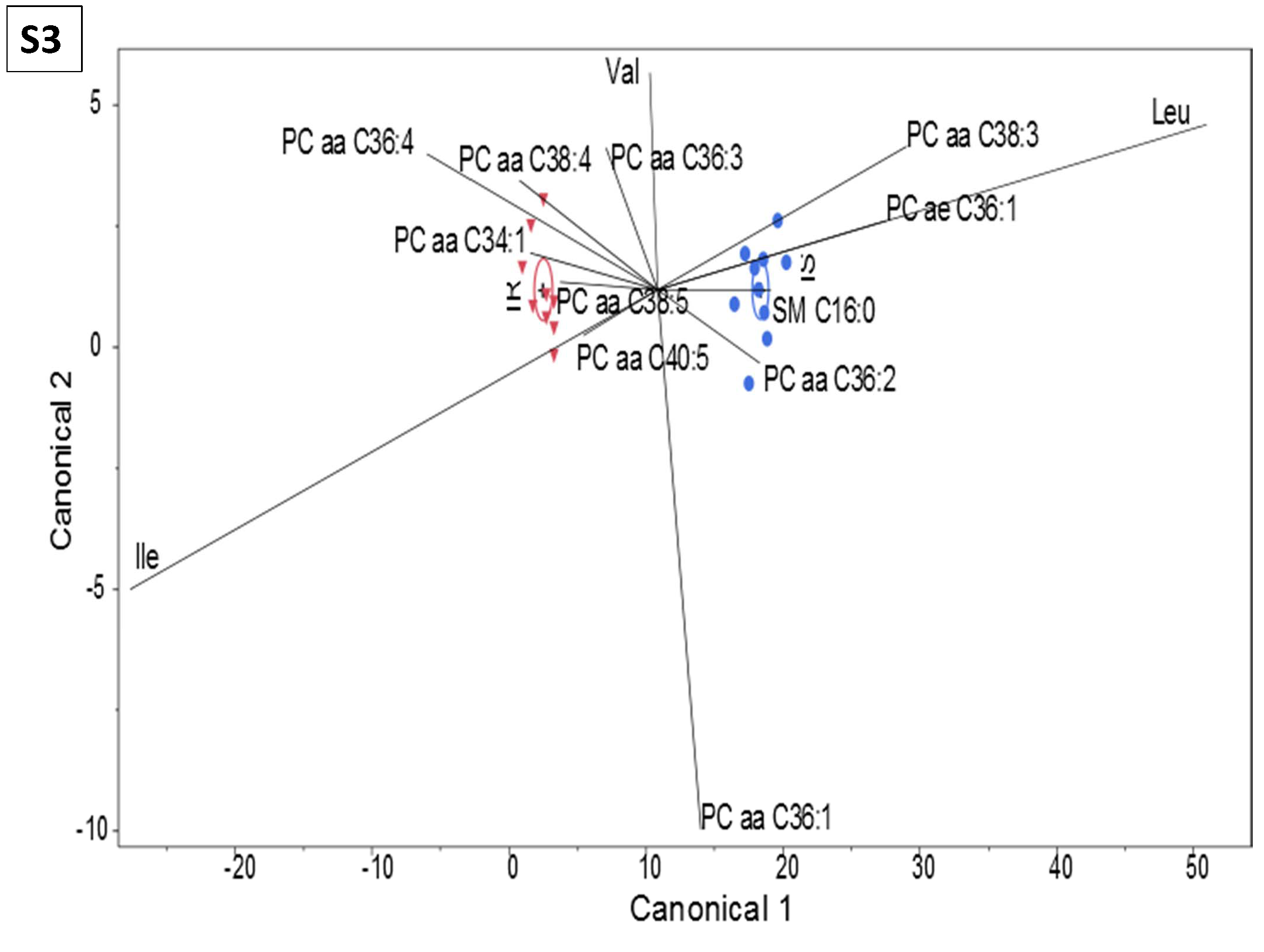

Supplement: Figure S3 — Discriminant Analysis of extracellular milieu. (TIFF) [file pone.0093148.s003.tiff]

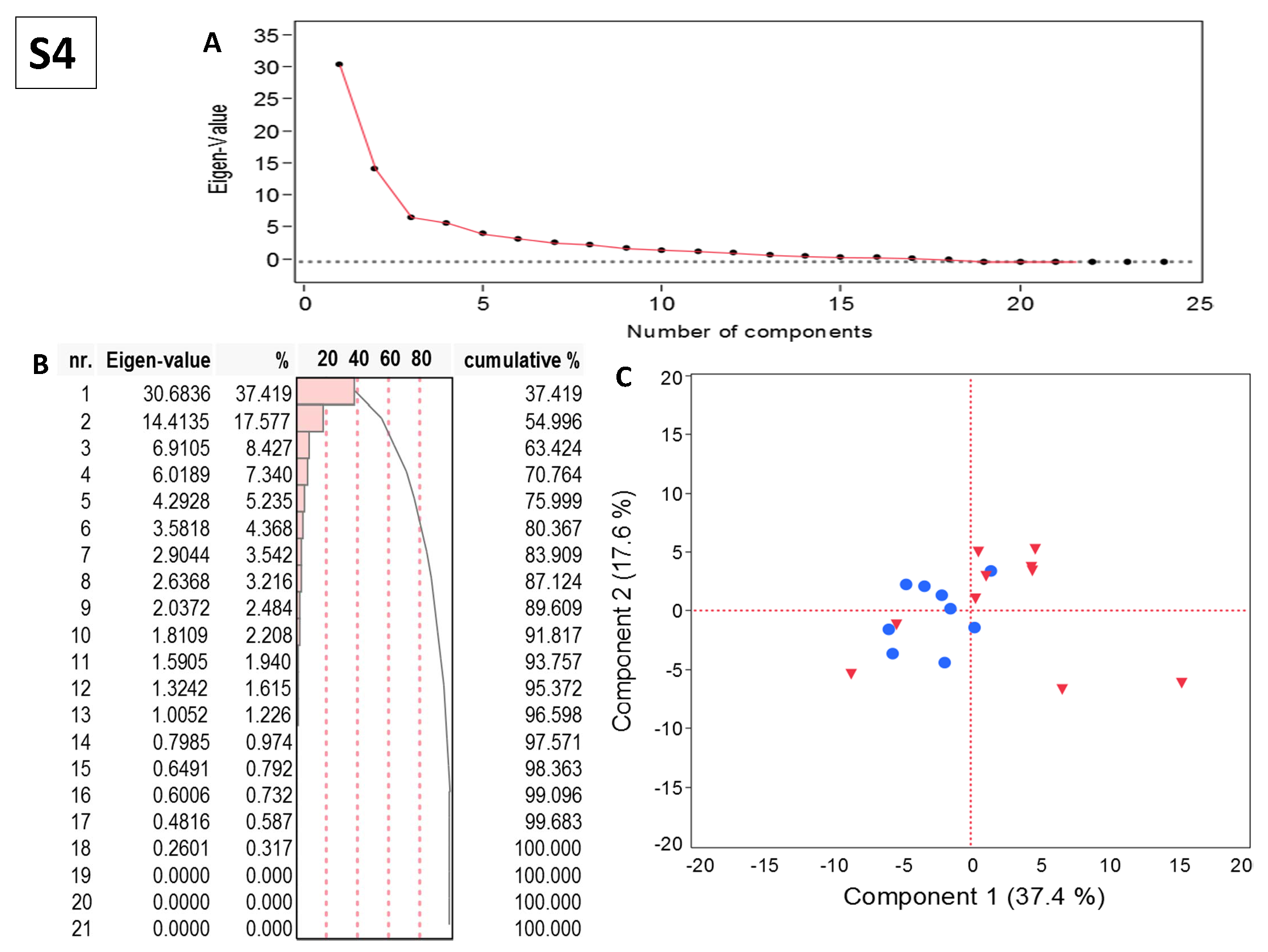

Supplement: Figure S4 — Graphical inter-group description intracellularly. (TIFF) [file pone.0093148.s004.tiff]

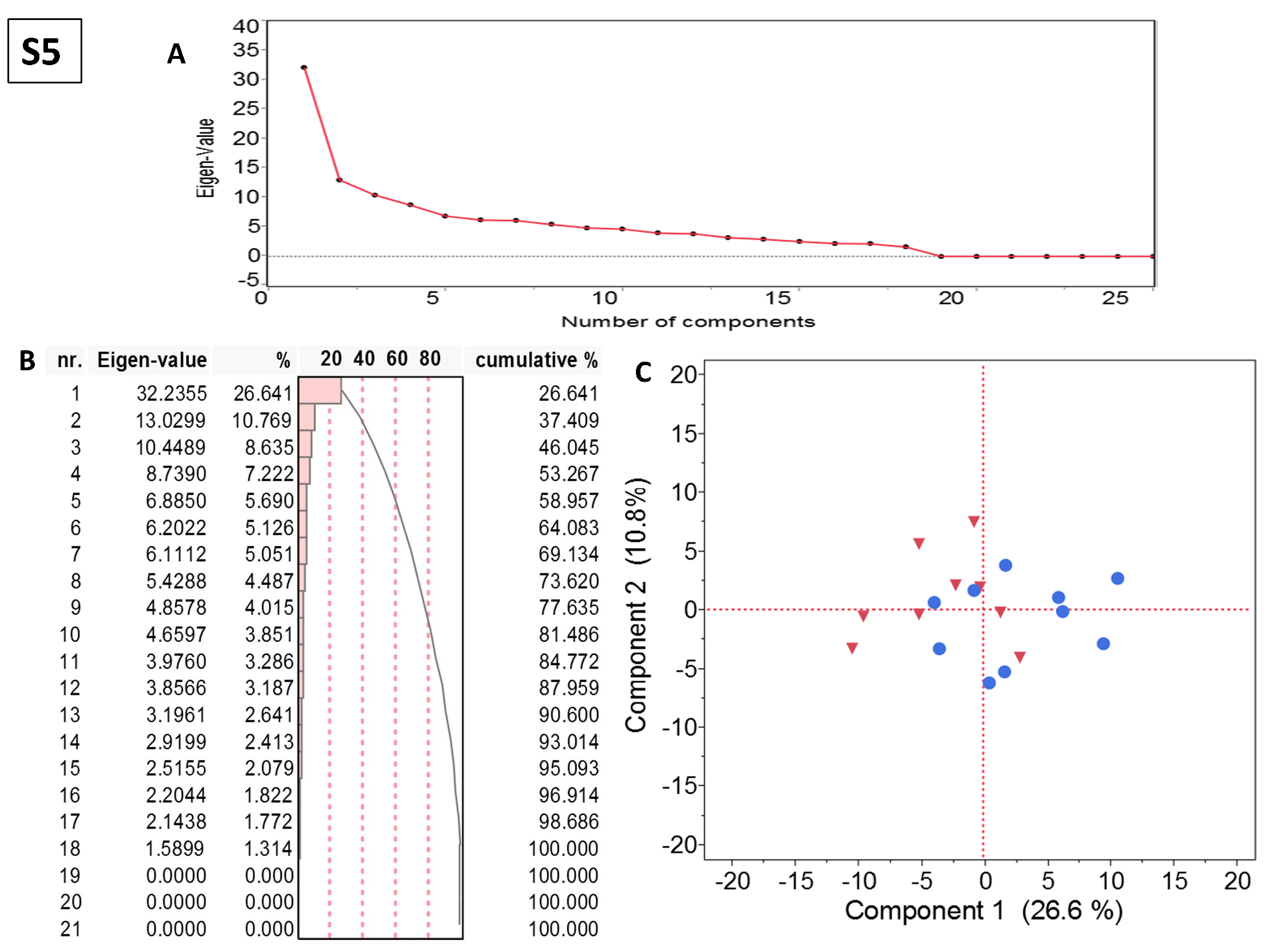

Supplement: Figure S5 — Graphical inter-group description extracellularly. (TIFF) [file pone.0093148.s005.tiff]

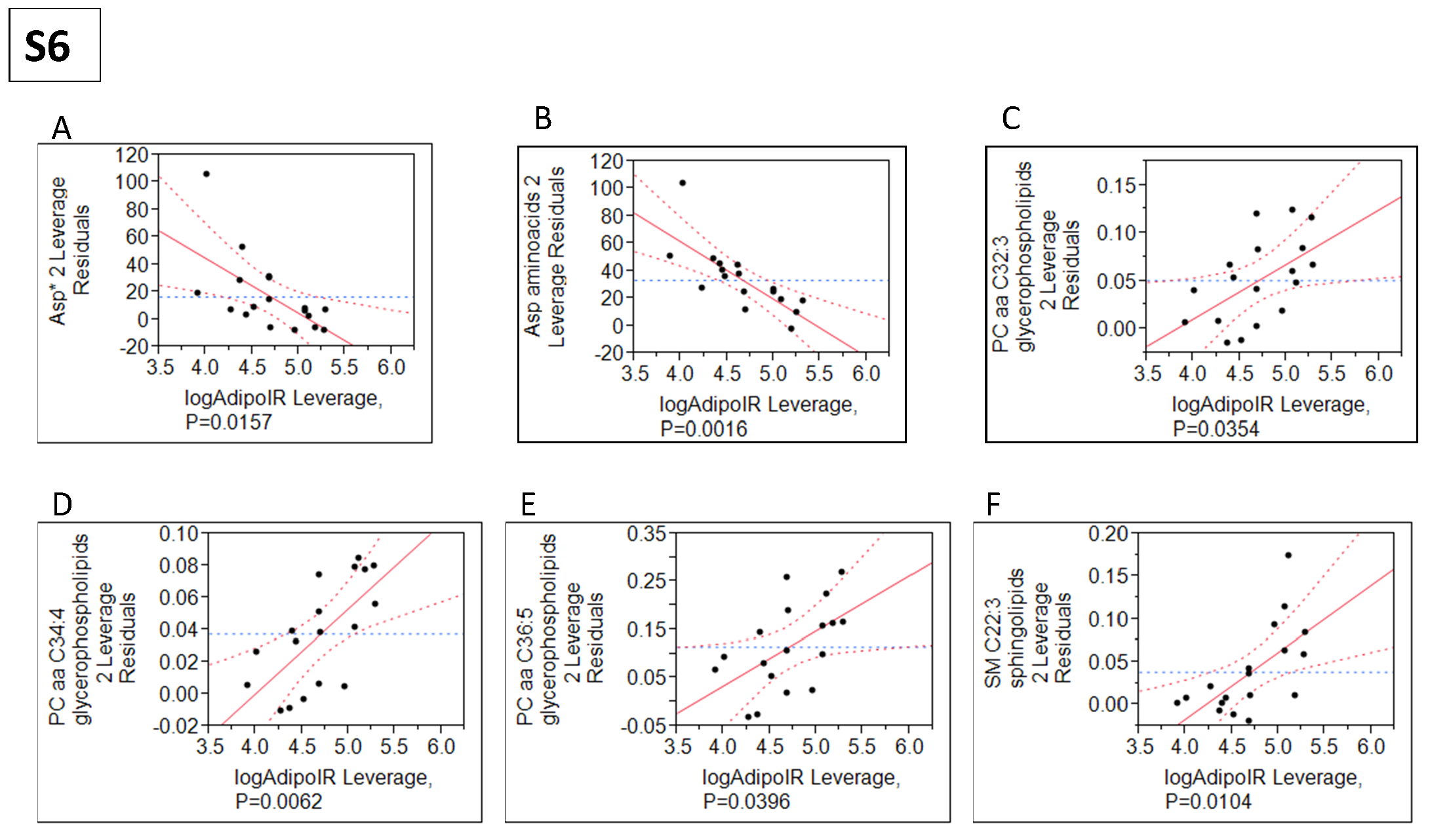

Supplement: Figure S6 — Correlation of metabolites with AdipoIR index. (TIFF) [file pone.0093148.s006.tiff]
