# Supplementary material for: Metabolic Signatures of Cultured Human Adipocytes from Metabolically Healthy versus Unhealthy Obese Individuals
Source: PLoS One. 2014 Apr 2;9(4):e93148. doi: 10.1371/journal.pone.0093148 (PMC3973696; doi:10.1371/journal.pone.0093148)
Supplement: File S1 — File includes Supplementary Research Design and Methods, Supplemental References, legends for Figures S1-S6, and Tables S1–S3. (DOCX) [file pone.0093148.s007.docx]

**File S1**

**Electronic Supplementary Material**

*Supplementary Research Design and Methods*

**Phenotyping:** Tests all were performed after an overnight fast. Insulin levels were measured on ADVIA Centaur XP, routine laboratory tests performed on ADVIA 1800 clinical chemistry system (Siemens healthcare systems, Erlangen, Germany). Serum plasminogen activator inhibitor (PAI) -1 (BioVendor, Heidelberg, Germany), fetuin-A, leptin, adiponectin (R&D systems, Wiesbaden, Germany), and sex hormone-binding globulin (SHBG) (Hölzel Diagnostika, Köln, Germany) were measured by ELISAs. Percentage of body fat was measured by bioelectrical impedance (BIA-101, RJL Systems, Detroit, USA). Adipose Insulin Resistance index (Adipo-IR index) was calculated as suggested by e.g. [[1](#_ENREF_1)], FFA x fasting insulin.

**Isolation and culture of human adipocytes:** If not otherwise indicated, media, trypsin and saline were purchased from Lonza, Basel, Switzerland. Cell culture plastic was bought from TPP, Trasadingen, Switzerland. Isolated preadipocytes were seeded in 15cm dishes in growth medium [Ham’s F12/α-MEM (1:1)/20% FCS/1% chicken embryo extract (Seralab, UK)/2mM glutamine/0.1mg/ml Pen-Strep, 0.5µg/ml Amphotericin B] with medium exchange three times a week. At 70-80% confluence, cells were split by trypsination and seeded in 6-well-plates with a density of 2,000 cells/cm^2^ and 2ml medium volume/well. The homogeneity of the culture was controlled microscopically. The cells were grown up to optical confluence plus 2 days, then transferred to basal medium [containing DMEM/F12 (1:1)/5% FCS/2mM glutamine/0.1mg/ml Pen-Strep, 0.5µg/ml Amphotericin B/17µM pantothenic acid/1µM biotin/2 µg/ml apo-transferrin/1µM human insulin (Roche Diagnostics, Grenzach, Germany)/1µM dexamethasone (Sigma Aldrich, München, Germany)/10µM troglitazone (Sigma Aldrich)] plus 0.5mM isobutylmethylxanthine (Sigma Aldrich),2nM triiodo-thyronine (Sigma Aldrich) and 50µM indomethacin (Sigma Aldrich) for 7 days; medium was changed every second day. Then, the preadipocytes were terminally differentiated until day 20 in basal medium alone, medium exchange every second/third day.

**Metabolic analysis:** *Measurement of free fatty acids:* the content of individual free fatty acids in the samples have been determined as their corresponding methyl ester derivatives (FAME’s) using gas chromatography coupled with mass spectrometric detection (Agilent 7890 GC / 5975 MSD) after derivatization. Samples were treated with methanolic HCl solution for a prolonged time period to completely convert free fatty acids into their methyl esters representing the free fatty acid (FFA) content. Chromatograms in Selected Ion Monitoring mode with four characteristic ions were recorded for quantitation of individual FAME’s. External standard calibration curves were used to calculate the corresponding concentrations. Unknown FAME’s (external standards not available) were identified using a combination of spectra recorded in SCAN mode, respective ratios of characteristic ions and the retention behavior. Their (semi)-quantification was carried out with response factors extra- and/or intra-polated from the nearby eluting compounds having the same number of double bonds. *Eicosanoids and oxidized fatty acids (prostaglandins):* eicosanoids and other oxidized polyunsaturated fatty acids (PUFAs) were extracted from samples with aqueous acetonitrile that contained deuterated internal standards. The metabolites were determined by HPLC tandem mass spectrometry (LC-MS/MS) with Multiple Reaction Monitoring (MRM) in negative mode using a SCIEX API 4000 QTrap™ mass spectrometer with electrospray ionization. The LC-MS/MS method used for the analytical determination of eicosanoids has been published elsewhere [[2](#_ENREF_2)]. *Energy metabolism intermediates assay:* for the quantitative analysis of energy metabolism intermediates in the samples (glycolysis, citrate cycle, pentose phosphate pathway, urea cycle), hydrophilic interaction liquid chromatography-electro spray-MS/MS method in highly selective negative MRM detection mode was used. The MRM detection was performed using a SCIEX 4000 QTrap™ tandem mass spectrometry instrument (Applied Biosystems/MDS Analytical Technologies). The sample was protein precipitated and extracted simultaneously with aqueous methanol in a 96 well plate format. Internal standards (ratio external to internal standard) and external calibration were used for highly accurate quantitation.

**Supplemental references:**

1. Gastaldelli A, Harrison SA, Belfort-Aguilar R, Hardies LJ, Balas B, et al. (2009) Importance of changes in adipose tissue insulin resistance to histological response during thiazolidinedione treatment of patients with nonalcoholic steatohepatitis. Hepatology 50: 1087-1093.

2. Unterwurzacher I, Koal T, Bonn GK, Weinberger KM, Ramsay SL (2008) Rapid sample preparation and simultaneous quantitation of prostaglandins and lipoxygenase derived fatty acid metabolites by liquid chromatography-mass spectrometry from small sample volumes. Clinical chemistry and laboratory medicine : CCLM / FESCC 46: 1589-1597.

**Supplemental figures:**

**Figure S1. In vitro differentiated adipocytes.** OilRedO **(A)** - and NileRed/DAPI **(B)** staining.

**Figure S2.** **Discriminant Analysis of intracellular metabolites.** Conducted with thirteen metabolites detected by PCA. The group affiliation was predicted correctly. Axes reflect the two dimensions that best separate the groups, circles display multivariate means of the groups. Size of the circle corresponds to 95% confidence limit; groups that are significantly different tend to have non-intersecting circles.

**Figure S3. Discriminant Analysis of extracellular milieu.** Fourteen metabolites, detected by the PCA. The group affiliation was predicted correctly. Axes reflect the two dimensions that best separate the groups, circles display multivariate means of the groups. Size of the circle corresponds to a 95% confidence limit; groups that are significantly different tend to have non-intersecting circles.

**Figure S4**. **Graphical inter-group description intracellularly. (A)** scree-plot; the knee is at component 3. **(B)** shows the rapidly decreasing Eigen-values, thus, only the first two components (Eigen-values more than ten) were taken into account for PCA (fig. SM4C). **(C)** PCA; two groups are visible: IS (points) vs. IR (triangles). The percentage reflects the covered variance by a component, e.g., component 1 mirrors 37.4% of the variance of all data.

**Figure S5**. **Graphical inter-group description extracellularly.** (A) scree-plot of conditioned media; the knee is at component 2. **(B)** shows the corresponding Eigen-Values; Eigen-Values more than ten were taken into account for PCA (fig. SM5C). **(C)** PCA; the groups are: IS (points) vs. IR (triangles). The percentage reflects the covered variance by a component, e.g., component 1 mirrors 26.6% of the variance of all data.

**Figure S6. Correlation of metabolites with AdipoIR index.** Leverage plots of (A) aspartate, (B) aspartate (second kit), (C) PC aa 32:3, (D) PC aa 34:4, (E) PC aa 36:5, (F) SM C22:3. All adjusted for gender, age, and BMI; (A)-(E): intracellulary, (F): extracellular milieu.

**Supplemental tables:**

**Table S1. Intracellular metabolites used for the multivariate analyses, measured by p180 kit.**

| C0  acylcarnitines | lysoPC a C16:0 glycerophospholipids | PC aa C36:5 glycerophospholipids | PC ae C38:4 glycerophospholipids |
| --- | --- | --- | --- |
| C2  acylcarnitines | lysoPC a C16:1 glycerophospholipids | PC aa C36:6 glycerophospholipids | PC ae C38:5 glycerophospholipids |
| C3  acylcarnitines | lysoPC a C17:0 glycerophospholipids | PC aa C38:3 glycerophospholipids | PC ae C40:5 glycerophospholipids |
| C3-OH  acylcarnitines | lysoPC a C18:0 glycerophospholipids | PC aa C38:4 glycerophospholipids | PC ae C42:3 glycerophospholipids |
| C4  acylcarnitines | lysoPC a C18:1 glycerophospholipids | PC aa C38:5 glycerophospholipids | PC ae C44:3 glycerophospholipids |
| C9  acylcarnitines | lysoPC a C20:4 glycerophospholipids | PC aa C38:6 glycerophospholipids | PC ae C44:4 glycerophospholipids |
| Ala  aminoacids | PC aa C28:1 glycerophospholipids | PC aa C40:3 glycerophospholipids | PC ae C44:5 glycerophospholipids |
| Arg  aminoacids | PC aa C30:0 glycerophospholipids | PC ae C30:1 glycerophospholipids | SM (OH) C14:1 sphingolipids |
| Gly  aminoacids | PC aa C30:2 glycerophospholipids | PC ae C32:1 glycerophospholipids | SM (OH) C16:1 sphingolipids |
| His  aminoacids | PC aa C32:0 glycerophospholipids | PC ae C32:2 glycerophospholipids | SM (OH) C22:1 sphingolipids |
| Ile  aminoacids | PC aa C32:1 glycerophospholipids | PC ae C34:0 glycerophospholipids | SM (OH) C22:2 sphingolipids |
| Lys  aminoacids | PC aa C32:2 glycerophospholipids | PC ae C34:1 glycerophospholipids | SM C16:0  sphingolipids |
| Orn  aminoacids | PC aa C32:3 glycerophospholipids | PC ae C34:2 glycerophospholipids | SM C16:1  sphingolipids |
| Phe  aminoacids | PC aa C34:1 glycerophospholipids | PC ae C34:3 glycerophospholipids | SM C18:0  sphingolipids |
| Pro  aminoacids | PC aa C34:2 glycerophospholipids | PC ae C36:1 glycerophospholipids | SM C18:1  sphingolipids |
| Ser  aminoacids | PC aa C34:3 glycerophospholipids | PC ae C36:2 glycerophospholipids | SM C24:0  sphingolipids |
| Thr  aminoacids | PC aa C34:4 glycerophospholipids | PC ae C36:3 glycerophospholipids | SM C24:1  sphingolipids |
| Trp  aminoacids | PC aa C36:1 glycerophospholipids | PC ae C36:4 glycerophospholipids | SM C26:1  sphingolipids |
| Tyr  aminoacids | PC aa C36:2 glycerophospholipids | PC ae C36:5 glycerophospholipids | H1  sugars |
| Val  aminoacids | PC aa C36:3 glycerophospholipids | PC ae C38:0 glycerophospholipids |  |
| Creatinine  biogenic amines | PC aa C36:4 glycerophospholipids | PC ae C38:3 glycerophospholipids |  |

**Table S2. Extracellular metabolites used for the multivariate analyses, measured by p180 kit.**

| C14:1  acylcarnitines | Creatinine  biogenic amines | PC aa C36:5 glycerophospholipids | PC ae C38:5 glycerophospholipids |
| --- | --- | --- | --- |
| C14:2-OH acylcarnitines | Kynurenine  biogenic amines | PC aa C36:6 glycerophospholipids | PC ae C38:6 glycerophospholipids |
| C16:1-OH acylcarnitines | Met-SO  biogenic amines | PC aa C38:1 glycerophospholipids | PC ae C40:2 glycerophospholipids |
| C2  acylcarnitines | Sarcosine  biogenic amines | PC aa C38:3 glycerophospholipids | PC ae C40:3 glycerophospholipids |
| C3  acylcarnitines | Serotonin  biogenic amines | PC aa C38:4 glycerophospholipids | PC ae C40:5 glycerophospholipids |
| C3-OH  acylcarnitines | Taurine  biogenic amines | PC aa C38:5 glycerophospholipids | PC ae C40:6 glycerophospholipids |
| C4  acylcarnitines | total DMA  biogenic amines | PC aa C38:6 glycerophospholipids | PC ae C42:1 glycerophospholipids |
| C5  acylcarnitines | lysoPC a C16:0 glycerophospholipids | PC aa C40:2 glycerophospholipids | PC ae C42:2 glycerophospholipids |
| C5-OH (C3-DC-M) acylcarnitines | lysoPC a C16:1 glycerophospholipids | PC aa C40:3 glycerophospholipids | PC ae C42:3 glycerophospholipids |
| Ala  aminoacids | lysoPC a C17:0 glycerophospholipids | PC aa C40:4 glycerophospholipids | PC ae C44:3 glycerophospholipids |
| Arg  aminoacids | lysoPC a C18:0 glycerophospholipids | PC aa C40:5 glycerophospholipids | PC ae C44:4 glycerophospholipids |
| Asn  aminoacids | lysoPC a C18:1 glycerophospholipids | PC aa C42:1 glycerophospholipids | PC ae C44:5 glycerophospholipids |
| Cit  aminoacids | lysoPC a C18:2 glycerophospholipids | PC aa C42:2 glycerophospholipids | SM (OH) C14:1 sphingolipids |
| Gln  aminoacids | lysoPC a C20:3 glycerophospholipids | PC aa C42:4 glycerophospholipids | SM (OH) C16:1 sphingolipids |
| Gly  aminoacids | lysoPC a C20:4 glycerophospholipids | PC ae C30:1 glycerophospholipids | SM (OH) C22:1 sphingolipids |
| His  aminoacids | PC aa C28:1 glycerophospholipids | PC ae C32:1 glycerophospholipids | SM (OH) C22:2 sphingolipids |
| Ile  aminoacids | PC aa C30:0 glycerophospholipids | PC ae C32:2 glycerophospholipids | SM (OH) C24:1 sphingolipids |
| Leu  aminoacids | PC aa C30:2 glycerophospholipids | PC ae C34:0 glycerophospholipids | SM C16:0  sphingolipids |
| Lys  aminoacids | PC aa C32:0 glycerophospholipids | PC ae C34:1 glycerophospholipids | SM C16:1  sphingolipids |
| Met  aminoacids | PC aa C32:1 glycerophospholipids | PC ae C34:2 glycerophospholipids | SM C18:0  sphingolipids |
| Orn  aminoacids | PC aa C32:2 glycerophospholipids | PC ae C34:3 glycerophospholipids | SM C18:1  sphingolipids |
| Phe  aminoacids | PC aa C32:3 glycerophospholipids | PC ae C36:1 glycerophospholipids | SM C20:2  sphingolipids |
| Pro  aminoacids | PC aa C34:1 glycerophospholipids | PC ae C36:2 glycerophospholipids | SM C22:3  sphingolipids |
| Ser  aminoacids | PC aa C34:2 glycerophospholipids | PC ae C36:3 glycerophospholipids | SM C24:0  sphingolipids |
| Trp  aminoacids | PC aa C34:3 glycerophospholipids | PC ae C36:4 glycerophospholipids | SM C24:1  sphingolipids |
| Tyr  aminoacids | PC aa C34:4 glycerophospholipids | PC ae C36:5 glycerophospholipids | SM C26:0  sphingolipids |
| Val  aminoacids | PC aa C36:0 glycerophospholipids | PC ae C38:0 glycerophospholipids | SM C26:1  sphingolipids |
| Ac-Orn  biogenic amines | PC aa C36:1 glycerophospholipids | PC ae C38:1 glycerophospholipids | H1  sugars |
| ADMA  biogenic amines | PC aa C36:2 glycerophospholipids | PC ae C38:2 glycerophospholipids |  |
| alpha-AAA  biogenic amines | PC aa C36:3 glycerophospholipids | PC ae C38:3 glycerophospholipids |  |
| Carnosine  biogenic amines | PC aa C36:4 glycerophospholipids | PC ae C38:4 glycerophospholipids |  |

**Table S3.Analyzed metabolite ratios in extracellular milieu**

| aromatic AA | essential AA | ornithine/arginine | total SM-OH |
| --- | --- | --- | --- |
| ADMA/arginine | Fisher ratio | putrescine/ornithine | tyrosine/phenylalanine |
| BCAA | glucogenic AA | SDMA/arginine |  |
| citrulline/arginine | kynurenine/tryptophan | serotonin/tryptophan |  |
| citrulline/ornithine | met-SO/methionine | total DMA/arginine |  |

AA: amino acids; (A)DMA: (asymmetrically) dimethylated arginine; SDMA: symmetrically dimethylated arginine; BCAA: branched chain amino acids; Fisher ratio: BCAA/aromatic AA; met-SO: sulfoxidized methionine; SM-OH: hydroxysphingomyelins.
